# Did the International Trade in Crops Lead to Global Cropland Saving or Wasting in the Period 2000–2022?

**DOI:** 10.3390/foods13152371

**Published:** 2024-07-26

**Authors:** Tianbao Zhang, Qiyuan Hu, Tanglu Li, Xiang Gao, Yi Zhou, Xiaojie Liu, Fei Lun

**Affiliations:** 1 College of Land Science and Technology, China Agricultural University, Beijing 100193, China; 2021321010301@cau.edu.cn (T.Z.); qiyuan_hu@cau.edu.cn (Q.H.); s20233213591@cau.edu.cn (T.L.); b20223211040@cau.edu.cn (X.G.); 2 School of Geographical Sciences, Hunan Normal University, Changsha 410081, China; z20126933@163.com; 3 Key Laboratory of Natural Resource Coupling Process and Effects, Institute of Geographic Sciences and Natural Resources Research, Chinese Academy of Sciences, Beijing 100101, China; liuxj@igsnrr.ac.cn

**Keywords:** international crop trade, virtual cropland trade, cropland footprint, cropland use efficiency, cropland saving or wasting

## Abstract

The international food trade is beneficial for enhancing global food security but also raises issues such as global cropland redistribution, land use efficiency, and environmental problems. While current studies have examined the impacts of the international food trade on these issues, its long-term effects on global cropland use efficiency remain unclear, especially when considering different crops and countries. Utilizing the international trade theory and the principle of virtual cropland, this study explores the relationship between international food trade and global cropland use efficiency from 2000 to 2022. The results illustrate that the global crop trade surged by 142%, outpacing the 102% increase in virtual cropland trade, which was attributed to crop yield enhancements. By 2022, the global virtual cropland trade encompassed 10.7% of the total croplands, with China emerging as the foremost importer, particularly due to soybean imports. Notably, the global crop trade led to substantial cropland savings and higher cropland use efficiency, totaling 1244.9 million hectares (Mha) between 2000 and 2020. These gains were largely attributed to the superior yields of major crop-exporting countries. Despite these gains, socio-economically vulnerable countries face significant challenges, potentially compromising their food security amidst the complexities of the global trade dynamics.

## 1. Introduction

Population growth and dietary shifts have significantly increased the global food demand, projected to reach 30,000 million metric tons (Mt) by 2050 [1], a 50–60% increase from the 2019 levels [2,3]. This accelerating increase presents substantial challenges to food security, particularly in African countries where the domestic production is constrained by limited cropland [4]. Consequently, the United Nations has prioritized “Zero Hunger” as the second global Sustainable Development Goal (SDG, https://www.un.org/sustainabledevelopment/zh/hunger/ (accessed on 10 June 2024)), aiming to end hunger and achieve food security. 

Cropland, a critical but finite natural resource, is fundamental to food security. However, the global cropland area was limited to 1579.9 million hectares (Mha) in 2021. Significant disparities in cropland area and yield exist among countries due to differences in natural resources, climate, and socio-economic factors [5]. Some nations, especially impoverished ones, cannot ensure food security solely through domestic production. For instance, Venezuela and Syria have less than 0.12 ha of cropland per capita, significantly below the global average of 0.2 ha, and rank 106th and 113th out of the total 113 countries on the food security index [6]. Thus, it is crucial to enhance their food security [7]. Cropland expansion has boosted crop production but has also pushed the global land use beyond the sustainable planetary boundary [8], potentially jeopardizing the future sustainable development. Additionally, extensive cultivation in some countries leads to environmental issues like nitrogen imbalance [8]. Hence, it is imperative to undertake long-term investigations into global cropland dynamics, as well as its associated food security and other issues. 

International trade, leveraging advantages in natural resources or labor, can enhance the global production efficiency, mitigating domestic imbalances between crop production and consumption, as explained by the international trade theory [9,10]. This trade increases crop availability and diversity in each country [11,12]. The global international trade in crops has witnessed a steady increase from 240.3 Mt in 2000 to 582.2 Mt in 2022. While beneficial for global food security and achieving the SDG2, the international trade also redistributes natural resources and presents global challenges [5,13]. 

Previous studies have pointed out that the global virtual cropland trade can account for about 20.1% of the global total cropland [14]. Thus, the international crop food trade could greatly lead to a global cropland redistribution [15]. Meanwhile, changes in cropland area and yield in one country can leave significant impacts on the global cropland use efficiency (saving or wasting), particularly given the current intensification of the international crop trade [14]. For instance, the increasing export of soybean and oil palm has led to deforestation in South America and Southeast Asia, causing issues such as land use change, carbon emissions, and biodiversity losses [16,17]. 

Based on the principles of “cropland footprint” [18], recent studies have shed light on how the international trade in crops influences the global cropland use efficiency and other issues at both global and regional scales [19,20,21,22,23], as well as their driving factors (such as natural endowments, economic levels, and others) [24,25,26,27,28,29]. For example, Qiang et al. pointed out that the global virtual cropland trade has been increasing in recent years, mainly in developing countries [21]; Teame et al. illustrated that population, GDP, and geographic distance had great influences on the global crop trade, as well as on their virtual cropland trade [25]; Ali et al. found that virtual cropland trade would become more frequent due to the increasing use efficiency [28]. However, variations in yield and cropland area among crops and countries make the long-term impacts of the international trade in crops on the global cropland use efficiency unclear, especially when considering different crops. 

This study, grounded in the international trade theory and the concept of virtual cropland, aims to explore the impact of the international crop trade on the global cropland use efficiency from 2000 to 2022. According to the global crop trade matrix from FAOSTAT, we aimed (1) to analyze the trade dynamics of maize, wheat, rice, and soybeans over these 23 years, noting trends and disparities; (2) to estimate the virtual cropland trade among countries based on the cropland footprint.; (3) to assess the impact of the international trade in crops on the global cropland use efficiency, considering cropland saving or wasting and their dynamics over these years; (4) to discuss its implications for global food security and environmental issues. 

## 2. Materials and Methods

### 2.1. Data Source

This study considered the detailed international trade of four important food crops—wheat, maize, rice, and soybeans—across 254 countries from 2000 to 2022. Geographical and economic classifications of these countries and regions are provided in Table S1 (see Supplementary Materials). The primary data source was the FAO Detailed Matrix of Country Trade (https://www.fao.org/faostat/en/#data/TM (accessed on 10 June 2024)), which provides comprehensive information on importing and exporting countries as well as trade volumes for these crops. Importantly, these trade volumes were recorded from the perspective of the importing countries. To enhance our analysis, we also incorporated crop yield data, demographic statistics, and consumer data from the FAO database (https://www.fao.org/faostat/zh/#data (accessed on 10 June 2024)). This additional data allowed for a more comprehensive understanding of the trade dynamics and their implications on global food security and cropland use efficiency.

### 2.2. Methods

#### 2.2.1. Cropland Footprint

In this study, “Material Flow Analysis” and “Cropland Footprint Analysis” were used to quantitatively investigate the international crop trade as well as its impacts on the global cropland use efficiency. “Cropland Footprint” refers to the cropland area required to produce a certain amount of specific crops, considering the agricultural practices [28]; essentially, cropland footprint can represent a crop yield and can be calculated as follows:

$$F_{ik}=A_{ik}/Y_{ik}$$

where the cropland footprint (*F**_ik_***) represents the area of cropland required by country *i* to produce one unit of crop *k*, expressed in ha/t. In more detail, *A_ik_* is the total cropland area of *k*, while *Y_ik_* is the yield of crop *k*.

#### 2.2.2. Virtual Cropland Trade

To better understand the impacts of international trade, the concepts of virtual cropland and its trade are used here. “Virtual cropland” refers to the area of cropland required to produce traded crops by the exporting countries, and thus “virtual cropland trade” is defined as the traded volume of this “virtual cropland” from the exporting country to the importing country [15]. For one specific crop, its virtual cropland trade can be estimated by multiplying its traded volume by the cropland footprint of the exporting country [15]. The specific formula is as follows:

$$VLT=T_{i,j}\times F_{i}$$

where virtual cropland trade (*VLT*) represents the virtual cropland embedded in crop trade from country *i* to country *j* and is estimated by the volume of the traded crop *T**_i,j_*** and the cropland footprint *F**_i_*** of the exporting country *i*. In more detail, *T**_i,j_*** is the trade volume of one specific crop exported from country *i* to country *j*, measured in tons; *F**_k,i_*** is the cropland footprint, which means the area of crop land required by country *i* to produce one unit of crop, expressed in ha per ton.

#### 2.2.3. Global Cropland Use Efficiency Due to the International Crop Trade

Due to disparities in natural resources, agricultural practices, and socio-economic factors, the crop yields vary across different countries and crops. When crops are traded from countries with a low cropland footprint (high yield) to those with a high cropland footprint (low yield), less cropland is required by the exporting country to produce these traded crops, which leads to global cropland savings (namely, a high global cropland use efficiency). Conversely, when crops are traded from high-footprint countries to low-footprint countries, this trade results in global cropland wasting (i.e., a low global cropland use efficiency). Thus, the difference in virtual croplands between exporting and importing countries allows us to assess the global cropland saving or wasting resulting from the global international crop trade, referred to as global cropland use efficiency (GCUE), between the exporting country *i* and the importing country *j*. The specific formula is as follows:

$$GCUE=VLT_{I}-VLT_{E}=T_{i,j}\times F_{j}-T_{i,j}\times F_{i}$$

where *GCUE* represents the area of cropland saved (high efficiency) or wasted (low efficiency) when country *i* exports crop *k* to country *j*, measured in ha. When *GCUE* < 0, it indicates that the international crop trade has led to global cropland wasting; when *S* > 0, it indicates that international crop trade has contributed to global cropland savings. *VLT_I_* and *VLT_E_* are the virtual cropland from the perspectives of the importing country and the exporting country, and their equations are as follows:

$$VLT_{E}=\sum_{k=1}^{m}\sum_{i=1}^{n}T_{k,i,j}\times F_{k,i}$$

$$VLT_{I}=\sum_{k=1}^{m}\sum_{i=1}^{n}T_{k,i,j}\times F_{k,i}$$

*F**_k,_******_i_*** and *F**_k,_******_j_*** are the cropland footprint of crop *k* for the exporting country *i* and the importing country *j*. *T**_k,i,j_*** is the trade volume of crop *k* exported from country *i* to country *j*.

## 3. Results

### 3.1. International Crop Trade Dynamics between 2000 and 2022

**Figure 1:**
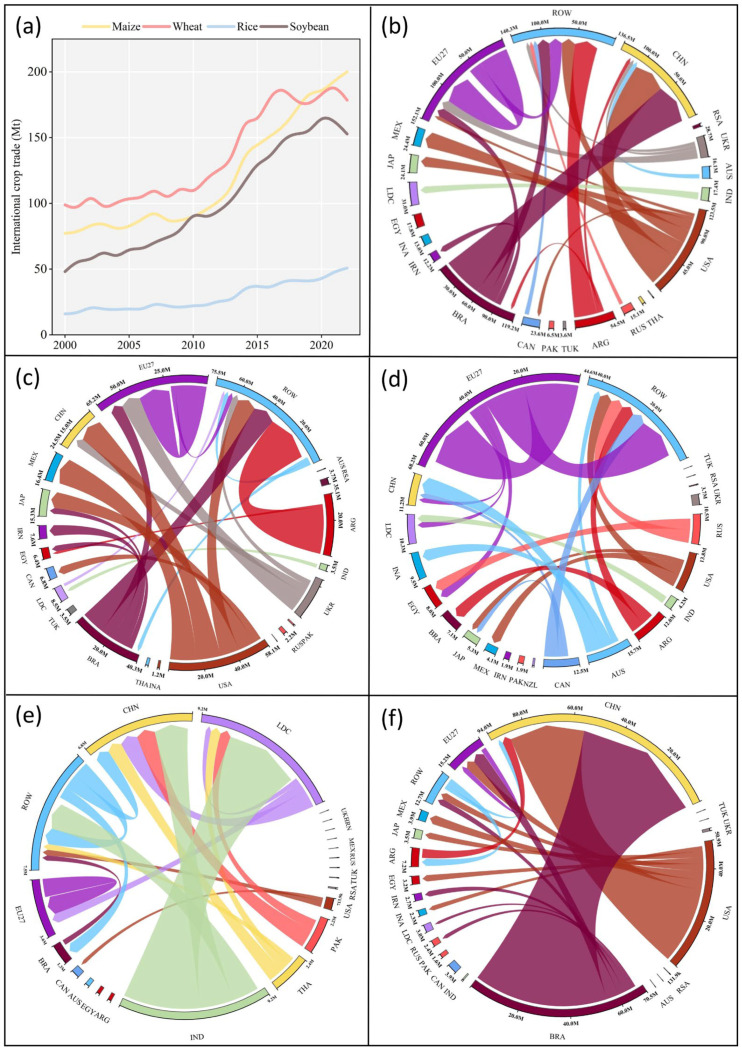
The international trade in crops among countries or regions between 2000 and 2022. (**a**) The total international trade volume of maize, wheat, rice, and soybean between 2000 and 2022; (**b**) the international trade of all four crops among countries in the year 2022, as well as of maize (**c**), wheat (**d**), rice (**e**), and soybean (**f**).

From 2000 to 2022, the global trade of maize, wheat, rice, and soybeans more than doubled, particularly before 2020 (Figure 1). However, the outbreak of the novel coronavirus epidemic and the Russia–Ukraine conflict hindered the increasing trajectory from 2020 to 2022. During these years, the global rice and soybean trades experienced a decline. By 2022, the trade volumes of maize, wheat, and soybeans hovered around 150–200 million metric tons (Mt) each, while that of rice lagged at 50.8 Mt. China, the E.U., and Mexico emerged as primary net food importers during this period, with notable increases in traded crops. China predominantly imported soybeans from the United States (U.S.A.) and Brazil, while the European Union (E.U.) and Mexico mainly sourced maize from Argentina and the U.S.A. The domestic crop production cannot meet the demands in China and Mexico, due to their large population; on the other hand, a significant number of crops were consumed for feeding livestock in the E.U. Thanks to their vast croplands and huge production, the U.S.A., Brazil, Australia, and Argentina assumed the roles of principal global exporters of soybeans (the former two countries), maize, and wheat.

The trade relationships between China and the U.S.A. and China and Brazil played increasingly pivotal roles in the global crop trade over the 23-year period, while the intra-European trade also saw significant growth. Some Least Developed Countries (LDCs), like Afghanistan, heavily relied on crop imports due to their large population and limited crop production. The Russia–Ukraine conflict has posed great challenges on their food security, as a significant decline has occurred in these countries for maize and wheat imports from Russia and Ukraine after the conflict started.

Notably, the global maize trade exhibited a substantial volume with relatively minor disparities among countries. The maize trade between the U.S.A. and Mexico constituted a significant portion and remained relatively stable over the past 23 years. The E.U. emerged as the largest wheat exporter, contributing 25% to the global wheat trade volume, with a significant portion directed towards LDCs in Africa. In contrast, rice was traded in smaller volumes and also concentrated in certain countries, like India. The Chinese soybean imports from the U.S.A. and Brazil increased due to the limited domestic production and dietary changes, accounting for 62% of the global soybean trade in 2022. However, the Sino–U.S. trade war caused a significant decline in the soybean trade between these countries after 2018, leading to increased soybean imports from Brazil to China.

### 3.2. The Cropland Footprint Trend for Crops and Countries between 2000 and 2022

**Figure 2:**
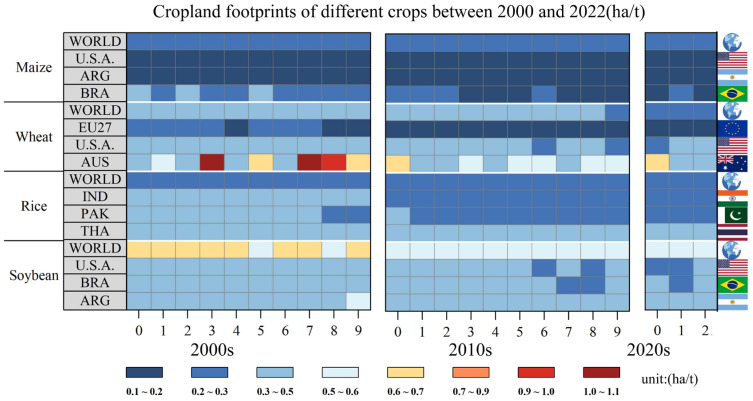
The cropland footprints of different crops, their global average and for three major exporting countries, between 2000 and 2022.

The cropland footprint varied significantly across countries and crops due to differences in natural endowments, agricultural practices, and socio-economic factors (Figure 2). The cropland footprints of the four crops exhibited a decreasing trend over the 23-year period due to advances in crop varieties and production methods. Maize kept its cropland footprint low due to higher yields compared to the other crops. For the three major maize-exporting countries, the United States, Brazil, and Argentina, the variation in the cropland footprint of maize was relatively small, ranging from 0.1 to 0.2 ha/t. The global cropland footprint of wheat showed a clear downward trend. Frequent weather extremes have had a significant impact on wheat production in Australia, particularly in 2007. The global rice cropland footprint remained relatively stable at 0.2–0.3 ha/t during these 23 years, but the three major rice-exporting countries of India, Pakistan, and Thailand presented higher cropland footprints than the global average level. This discrepancy is attributed to their relatively traditional rice cultivation practices, which heavily depend on local natural conditions. In contrast, the global soybean cropland footprint decreased by 28.6% over the same period, yet remained significantly high in 2022 compared to that of other major crops, being approximately 1.7 to 3.0 times higher. Additionally, the soybean cropland footprint of the three major soybean-exporting countries (the U.S.A., Brazil, and Argentina) was significantly lower than the global average level, indicating that their soybean exports could have positive impacts on the global cropland use efficiency.

### 3.3. Virtual Cropland Trade for Different Crops between 2000 and 2022

**Figure 3:**
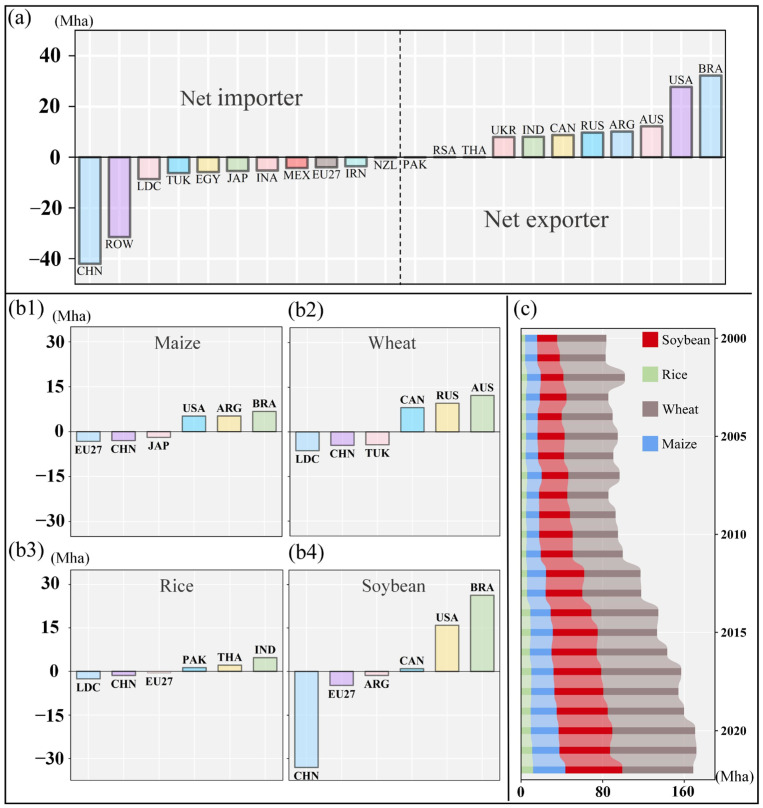
The virtual cropland trade for different crops between 2000 and 2022. (**a**) The major virtual cropland importers and exporters in 2022; (**c**) the global total virtual cropland trade for four crops between 2000 and 2022; (**b1**–**b4**) the major virtual cropland importers and exporters of maize (**b1**), wheat (**b2**), rice (**b3**), and soybean (**b4**) in 2022.

Between 2000 and 2020, the total global virtual cropland trade doubled from 83.4 million hectares (Mha) to 170.6 Mha (Figure 3). However, it decreased in the subsequent two years due to complex international situations. North America and South America, as primary crop-exporting regions, collectively accounted for 56.5% of the global virtual cropland exports. South America, driven by massive soybean and maize exports, gradually surpassed North America to become the largest virtual cropland exporter, with Brazil leading at 35.8 Mha of exported virtual cropland, comprising 54.2% of its total cropland. The U.S.A. and Argentina also witnessed large virtual cropland exports. In spite of crop trade increasing, the U.S.A. maintained relatively stable cropland exports at roughly 27.7 Mha, due to its increasing crop yield. With its rapid increase in rice exports, South Asia experienced a 12.2-fold increase in virtual cropland exports, reaching 8.7 Mha in 2022.

Europe emerged as a significant cropland importer, with the highest cropland import rate of 0.44 ha per capita, due to limited cropland and a developed livestock industry. East Asia, with its large population but limited cropland, had the largest share of virtual cropland imports, amounting to 52 Mha in 2022. Specifically, East Asia imported about 33.1 Mha of virtual soybean cropland in 2022, accounting for 19.6% of the global soybean cropland. China stood out as the largest importer of virtual cropland at 42.3 Mha in 2022, about 5.0 times the 2000 level, and this was mainly because of its huge soybean import. Despite some fluctuations, the LDCs imported substantial virtual croplands over the past 23 years, totaling 12.0 Mha in 2022, but this value was lower than that in 2020, influenced by complex international circumstances. 

The global virtual wheat cropland trade was the largest at 69.5 Mha in 2022, driven by a high trade volume but a low yield, with 10 countries exporting over 1.0 Mha of virtual wheat cropland each. The virtual cropland trade of soybean increased by 1.9 times due to the rapid growth of the global soybean trade, reaching 55.8 Mha in 2022. Brazil, the leading soybean exporter, totally exported approximately 26.7 Mha of virtual soybean croplands, nearly 5 times its 2000 level. While soybean expansion and exports brought economic benefits to Brazil, they also led to Amazon rainforest clearance. The Chinese virtual soybean imports surged to 33.1 Mha in 2022, accounting for 29.7% of the global traded virtual croplands. The global virtual rice cropland trade increased by 1.9 times, lagging behind the 3.6-fold increase in trade, thanks to yield improvements in major rice-exporting countries. The LDCs emerged as the major virtual rice cropland importers, with a 6.8-fold increase in 2022 compared to 2000, due to their significantly increased rice demands. Brazil was also the largest exporter of maize virtual cropland in 2022, followed by Argentina and the U.S.A. 

### 3.4. Global Cropland Saving or Wasting Due to the International Crop Trade between 2000 and 2022

**Figure 4:**
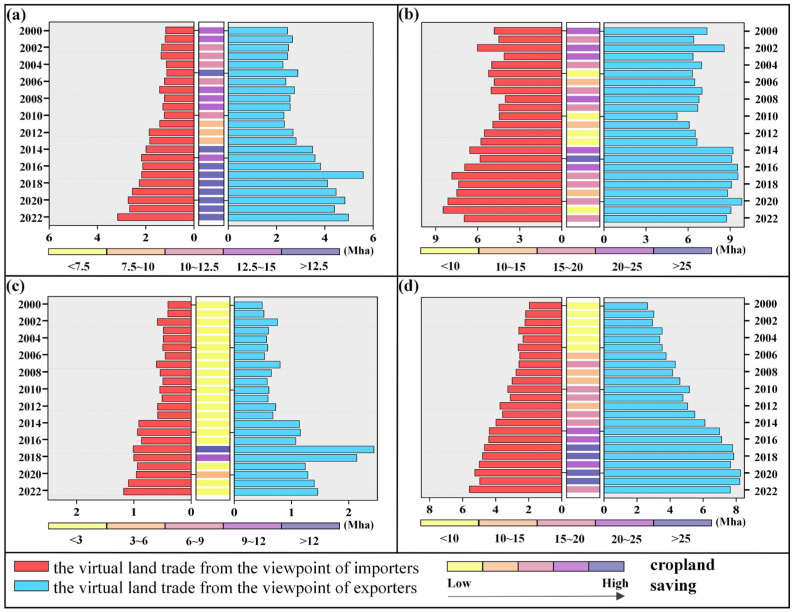
Global virtual cropland saving or wasting for different crops between 2000 and 2022. Maize (**a**), wheat (**b**), rice (**c**), and soybean (**d**).

The international crop trade has significantly enhanced the global cropland use efficiency, leading to substantial cropland savings from 2000 to 2022 (Figure 4). This positive trend was observed consistently for maize, wheat, rice, and soybean. Over the 23-year period, cumulative cropland savings totaled 1244.9 million hectares (Mha), representing approximately 79% of the global cropland. Soybean contributed the most cropland savings, corresponding to 424.0 Mha, followed by 421.8 Mha for wheat, 339.8 Mha for maize, and 59.4 Mha for rice. Therefore, the global crop trade was conducive to enhancing the global cropland use efficiency and promoting its sustainable cultivation. In 2022, the global international crop trade saved a total of 61 Mha of global cropland, a 1.32-fold increase compared to 2000. Soybean trade, benefiting from higher yields in the exporting countries, led to the largest cropland savings of 20.7 Mha in 2022, equivalent to 1.5% of the global soybean cropland. Both maize and wheat trades also contributed significantly to cropland savings, for over 18.0 Mha each in 2022. However, they exhibited different trends over these 23 years; specifically, the maize trade contributed to the annual cropland savings within the range of 8.7~21.7 Mha, whereas the wheat trade showed a decreasing contribution to the global cropland savings, from 25.4 Mha in 2000 to 18.0 Mha in 2022. Due to the similar rice yields between exporting and importing countries, the international rice trade resulted in relatively smaller cropland savings compared to the other three crops. Nevertheless, the global rice trade also exhibited a trend of increasing contribution to cropland savings, amounting to 2.3 Mha in 2022.

**Figure 5:**
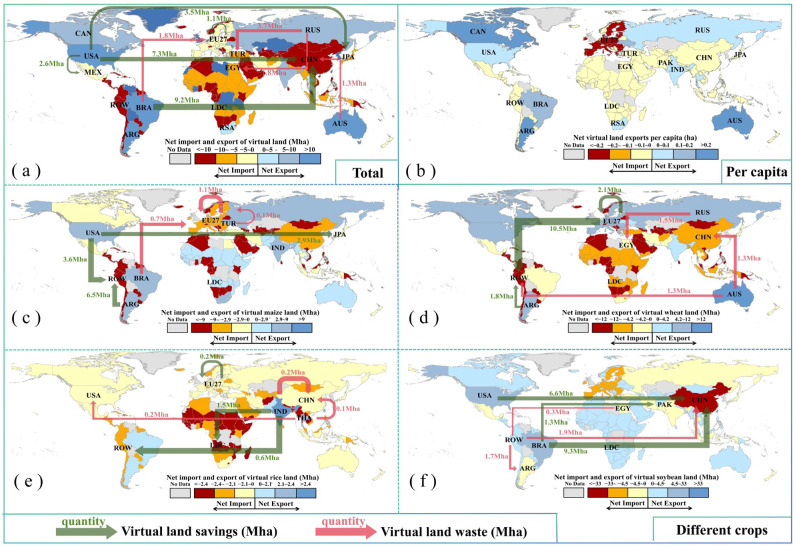
Global cropland saving or wasting due to the global crop trade for different crops. The total global cropland saving or wasting for the four crops (**a**), maize (**c**), wheat (**d**), rice (**e**), and soybean (**f**) in 2022. The net virtual cropland imports or exports per capita (**b**).

The global major crop exporters, such as the U.S.A., Brazil, and Argentina, are located in mid-latitude areas with favorable hydrothermal conditions and a flat terrain, which leads to high crop yields. Consequently, their crop exports significantly contribute to the global cropland savings (Figure 5). The E.U. countries, with their higher wheat yield, significantly saved the global wheat cropland, amounting to 10.5 Mha in 2022. In contrast, Australia’s extensive agricultural practices and vast croplands resulted in much lower wheat yields compared to other countries like China. As a result, the wheat trade from Australia to China led to a global cropland wastage. Enhancing the wheat yields in Australia could therefore improve the global food security. Brazil and the U.S.A., the top two soybean exporters, saved a total of 24.3 Mha of global soybean cropland in 2022, including 15.8 Mha saved through trade with China and 0.5 Mha saved through trade with the E.U. The U.S.A. and Argentina, benefiting from large flat plains and highly mechanized agricultural practices, have high maize yields, leading to substantial cropland savings of 10.1 Mha through the maize exports. Compared with countries in Africa, India exhibited a higher rice yield, and thus the international rice trade from India to LDCs and ROW could contribute to a total of 2.1 Mha cropland savings in 2022. However, the Indian rice yields were significantly lower than those in China, resulting in a rice cropland wastage of 0.2 Mha from trade between these two countries. Improving the rice yields in India through enhanced breeding technology and agricultural practices is crucial for global food security and cropland savings.

## 4. Discussion

### 4.1. Global and Regional Socio-Economic Circumstances, International Crop Trade, and Food Security

Over the past 23 years, factors such as El Niño, regional conflicts, epidemics, and other events impacted the global crop trade. Despite these challenges, the international crop trade strengthened and increased, resulting in significant global cropland savings and a higher cropland use efficiency. With limited cropland and poor natural resources but a large population, the LDCs heavily relied on the international crop trade and showed an upward trend in food security under relatively stable socio-economic conditions in recent years [7,29]. However, there was still approximately 19.2% of the population in LDCs under severe food shortage in 2019, compared to the global level of 9.2% [30]. In addition, these impoverished and undeveloped countries are highly sensitive to both global and local socio-economic changes, which can significantly impact their food security [7]. The outbreak of the COVID-19 pandemic in 2020 hindered the international crop trade, leading to a significant reduction in food availability and exacerbating the food shortages in these LDCs, resulting in serious malnutrition [2,31]. Previous studies indicated that global hunger nearly doubled by the end of 2020 as a result of the COVID-19 pandemic [32], with nearly 20% of the population affected in low- and middle-income countries. Moreover, regional conflicts and trade disputes can also significantly impact the global international crop trade. The Russia–Ukraine conflict has caused great turmoil in the global crop trade and has seriously influenced food security in Middle East, North Africa, and sub-Saharan Africa, which heavily relied on crop imports from Russia and Ukraine [26,33]. For example, Turkey saw its wheat imports from Russia drop by 15.5% after the conflict began, with negative effects on its food security. Additionally, other conflicts (such as economic disputes) could also alter the global crop trade matrix [34]. For instance, the Sino–U.S. trade war, which began in 2018, caused the soybean trade between the U.S.A. and China decline by 40%, while the soybean trade between Brazil and China strengthened, experiencing a 21% increase. In addition to the socio-economic conditions, natural disaster would also impact the global crop trade and global cropland use efficiency. For instance, the intensive El Niño events in 2003 and 2006–2007 led to a severe drought in Australia, causing a decline in wheat production. This natural disaster not only reduced the wheat exports from Australia, but also resulted in global cropland wasting due to the relatively lower crop yields. Thus, it is important to mitigate these negative impacts on international crop trade and global food security with regional cooperation, advanced technology transfer, and so on. 

### 4.2. International Crop Trade and Its Associated Environmental Issues

The rapid increase in international crop trade has the potential to enhance the global food security but also poses significant environmental challenges. These include issues related to water use, land use change, biodiversity loss, nutrient flows, and pollution [5,35]. With the growing consumption of animal products, both the E.U. and China have seen increased imports of soybeans from Brazil and other countries [36]. However, the need to meet the demand for soybean has led to Amazon rainforest clearance in Brazil and other South American countries. Da Silva et al. [37] highlighted the positive relationship between soybean cultivation and Amazon deforestation in Mato Grosso and Brazil. Given the critical role of the Amazon forest in maintaining global ecosystem services, such deforestation poses substantial environmental risks. Pendrill et al. [38] demonstrated that the international crop trade accounted for 39% of greenhouse gas emissions associated with tropical deforestation, underscoring the urgency of addressing the environmental impacts of the global soybean trade [39]. Further studies should be demanded to develop strategies to balance food security and Amazon rainforest protection. 

India, as the leading rice exporter, exported nearly 5 Mha of virtual rice cropland in 2022. However, its lower yield compared to those of importing countries like China has resulted in cropland wasting. Additionally, primitive crop practices in India have led to substantial greenhouse gas emissions and air pollution [40,41,42,43,44,45,46], causing serious disturbances to local ecosystems and incurring high economic costs [13,47,48,49,50]. During the prevalent period of rice residue burning, the PM2.5 concentration can reach 500 µg m^−3^ in Delhi [51], with environmental costs amounting to 8953 rubles per ha and a total value of 31.99 billion rubles in India [52]. Furthermore, straw burning in India also leads to nutrient losses, including 40% nitrogen loss, 33% potassium loss, and 45% sulfur loss [53,54]. Balancing rice yield improvement and environmental protection in India is crucial. Thus, for exporting countries, scientific, reasonable, and environment-friendly crop cultivation practices are conducive to enhancing the cropland sustainable utilization, increasing crop production as well as local and global environmental protection [55].

### 4.3. Limitations and Future Studies

Our studies investigated the impact of the international crop trade on the global cropland use efficiency between 2002 and 2022; however, some limitations still warrant attention. Firstly, our analysis focused on four major crops, i.e., maize, wheat, rice, and soybean. With dietary shifts, there is a notable increase in the global trade of other crops and animal products, which could also influence the global cropland redistribution and use efficiency. For example, the global barley trade increased by 12.1 Mt between 2000 and 2022, while the global poultry trade increased by 319.6%. Therefore, future studies should encompass a broader range of crops and animal products to provide a comprehensive understanding of the international trade’s impacts on the global cropland use efficiency. Additionally, our research primarily examined the influence of socio-economic conditions on the international food crop trade, but dietary shifts and population growth could also have some influences on the global crop trade. With the increase in animal products, particularly in developing countries, the soybean demands are projected to surge to twice the current level by 2050 [56,57], which could greatly influence the global cropland redistribution and use efficiency. Furthermore, the population growth trajectory will amplify the crop demands across the globe. Hence, future studies should investigate how dietary shifts and population increase will reshape the international crop trade as well as its associated issues in the future.

## 5. Conclusions

This study underscores the substantial impacts of the international crop trade on the global cropland use efficiency, showing positive effects overall. Over the period of 2000–2022, the global international crop trade experienced a significant growth, shaping a more intricate trade network influenced by various factors such as global and regional socio-economic conditions, natural resources, and crop yield. During this period, the cropland footprint for all four major crops showed a declining trend, although notable variations existed among them. Concurrently, the global virtual cropland trade doubled, reaching an equivalent of 10.7% of global cropland by 2022. China emerged as a pivotal player, becoming the largest importer of virtual cropland, primarily driven by increased soybean imports. The international crop trade has contributed to global cropland savings, displaying an upward trajectory over the 23-year period. The cumulative cropland savings amounted to 1244.9 Mha during this time, representing approximately 79% of the current global cropland area. Major crop exporters, endowed with favorable hydrothermal conditions and fertile lands, demonstrated higher yields, thus fostering the global cropland savings. However, impoverished and underdeveloped countries remained highly susceptible to both global and local socio-economic changes, which significantly impacted their food security. Future studies should adopt a broader scope, encompassing a wider array of crops and animal products, while also exploring the influence of dietary shifts and population dynamics on the patterns of the international crop trade.

## Data Availability

The original contributions presented in the study are included in the article/Supplementary Material, further inquiries can be directed to the corresponding author.

## Supplementary Materials

The following supporting information can be downloaded at: https://www.mdpi.com/article/10.3390/foods13152371/s1, Figure S1: The international crop trade among countries or regions. (a) The international trade of all four crops among countries in the year 2000; (b) the international trade of all four crops among countries in the year 2010; (c) the international trade of all four crops among countries in the year 2020; (d) the international trade of all four crops among countries in the year 2022; (e) the international rice trade among countries in the year 2022; (c) the international soybean trade among countries in the year 2022; Figure S2: The virtual cropland trade among countries or regions. (a) The international virtual cropland trade of all four crops among countries in the year 2000; (b) the international virtual cropland trade of all four crops among countries in the year 2010; (c) the international virtual cropland trade of all four crops among countries in the year 2020; (d) the international virtual cropland trade of all four crops among countries in the year 2022; Figure S3: The virtual maize land trade among countries or regions. (a) The international virtual maize land trade among countries in the year 2000; (b) the international virtual maize land trade among countries in the year 2022; Figure S4: The virtual wheat land trade among countries or regions. (a) The international virtual wheat land trade among countries in the year 2000; (b) the international virtual wheat land trade among countries in the year 2022; Figure S5: The virtual rice land trade among countries or regions. (a) The international virtual rice land trade among countries in the year 2000; (b) the international virtual rice land trade among countries in the year 2022; Figure S6: The virtual soybean land trade among countries or regions. (a) The international virtual soybean land trade among countries in the year 2000; (b) the international virtual soybean land trade among countries in the year 2022; Table S1: Geographical and economic classifications of the examined countries and regions.
