# Supplementary material for: Did the International Trade in Crops Lead to Global Cropland Saving or Wasting in the Period 2000–2022?
Source: Foods. 2024 Jul 26;13(15):2371. doi: 10.3390/foods13152371 (PMC11311825; doi:10.3390/foods13152371)
Supplement: Supplementary file 1 [file foods-13-02371-s001.zip › foods-3084673-supplementary.pdf]

International crop trade lead to global cropland saving or wasting during 2000-2022？

Tianbao Zhang [a], Qiyuan Hu [a]*, Tanglu Li [a], Xiang Gao [a], Yi Zhou [b], Xiaojie Liu [c], Fei Lun [a]*

[a] College of Land Science and Technology, China Agricultural University, Beijing 100193, China
[b] School of Geographical Sciences, Hunan Normal University, Changsha 410081, China
[c] Key Laboratory of Natural Resource Coupling Process and Effects, Institute of Geographic Sciences and Natural Resources Research, Chinese Academy of Sciences, Beijing 100101, China

Supplementary Figures

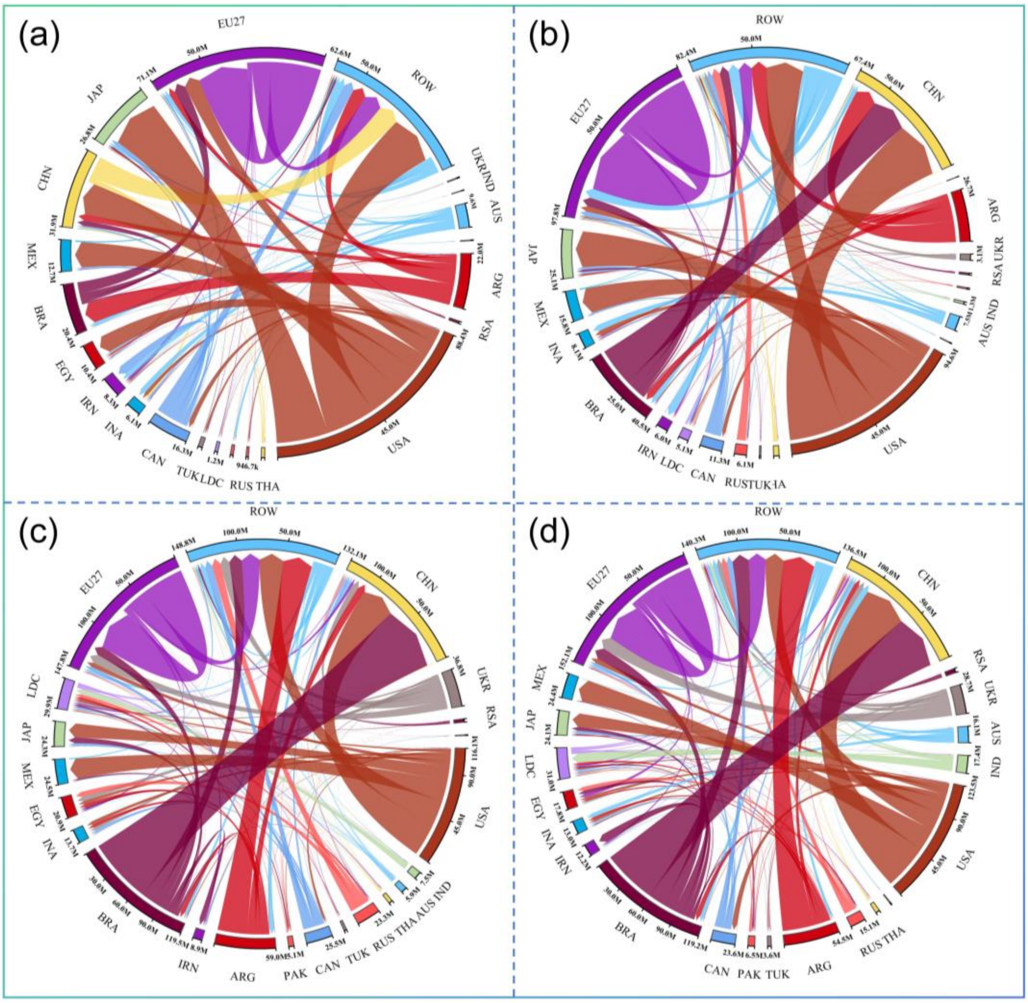

Figure S1: The international crop trades among countries or regions (a) The international trade of all four crops among countries in the year of 2000; (b) The international trade of all four crops among countries in the year of 2010; (c) The international trade of all four crops among countries in the year of 2020; (d) The international trade of all four crops among countries in the year of 2022; (e) The international rice trade among countries in the year of 2022; (c) The international soybean trade among countries in the year of 2022.

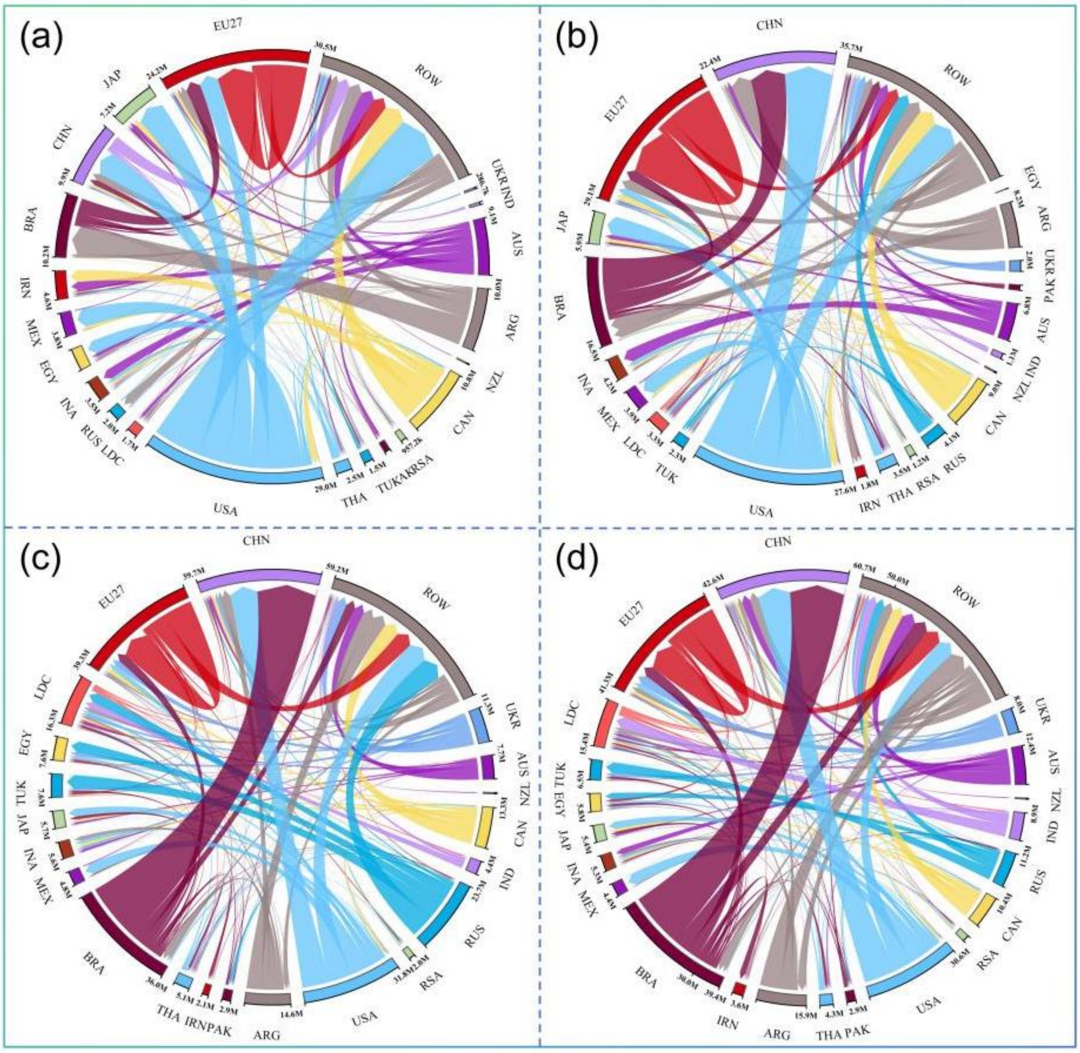

Figure S2: The virtual cropland trade among countries or regions (a) The international virtual cropland trade of all four crops among countries in the year of 2000; (b) The international virtual cropland trade of all four crops among countries in the year of 2010; (c) The international virtual cropland trade of all four crops among countries in the year of 2020; (d) The international virtual cropland trade of all four crops among countries in the year of 2022.

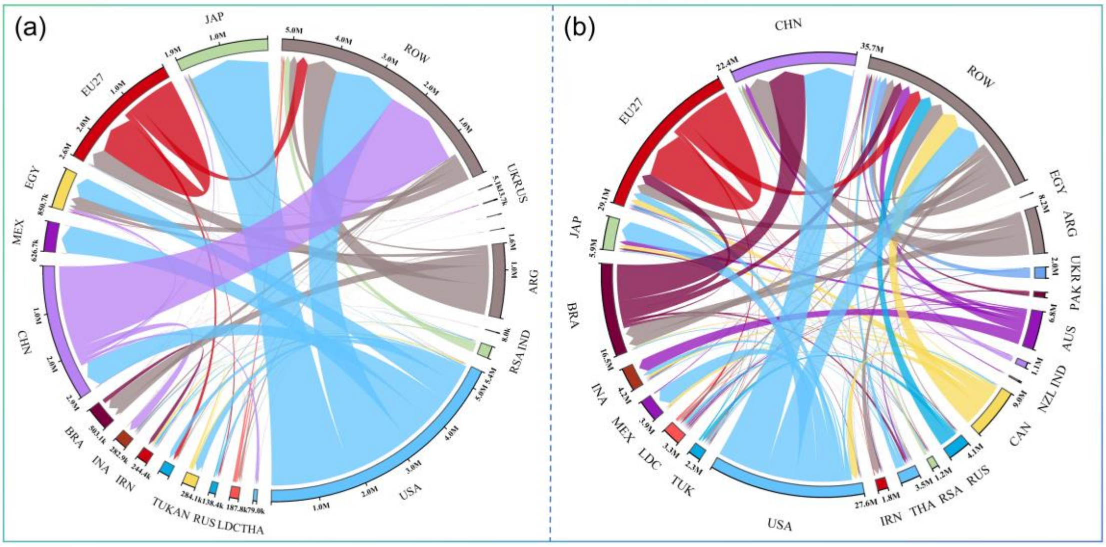

Figure S3: The virtual Maize land trade among countries or regions (a) The international virtual Maize land trade among countries in the year of 2000; (b) The international virtual Maize land trade among countries in the year of 2022.

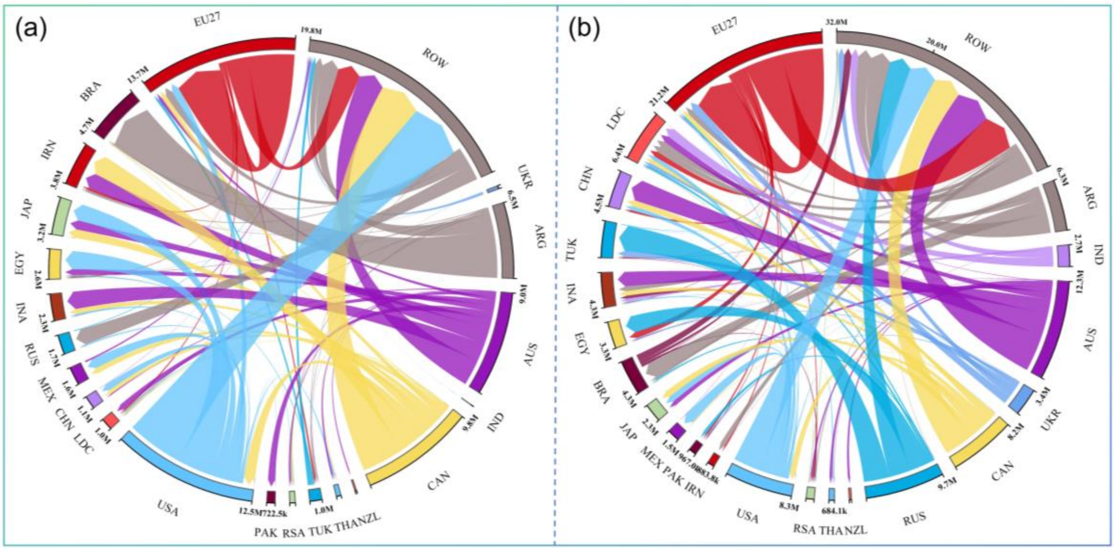

Figure S4: The virtual Wheat land trade among countries or regions (a) The international virtual Wheat land trade among countries in the year of 2000; (b) The international virtual Wheat land trade among countries in the year of 2022.

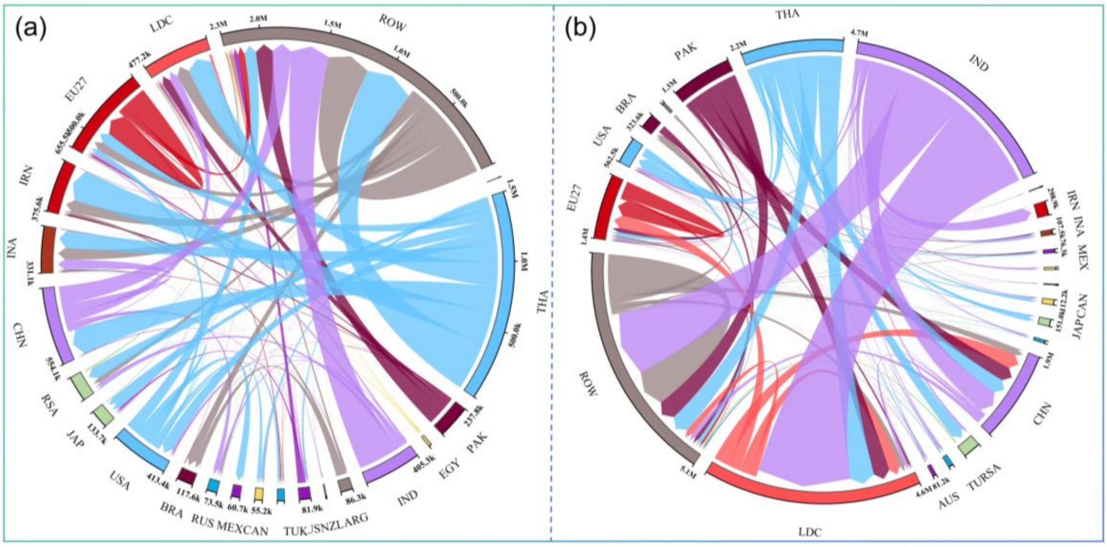

Figure S5: The virtual Rice land trade among countries or regions (a) The international virtual Rice land trade among countries in the year of 2000; (b) The international virtual Rice land trade among countries in the year of 2022.

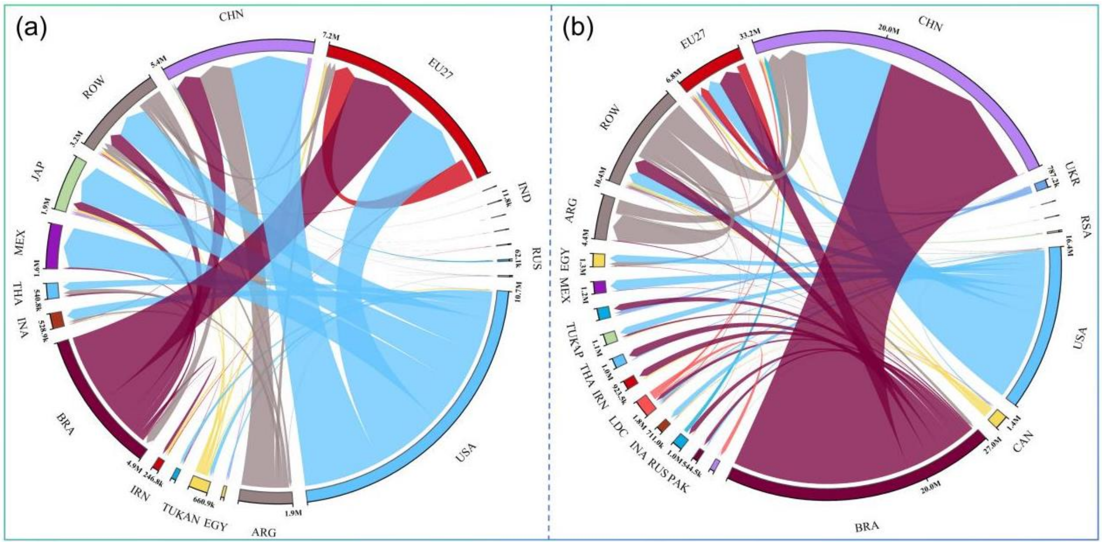

Figure S6: The virtual Soybean land trade among countries or regions (a) The international virtual Soybean land trade among countries in the year of 2000; (b) The international virtual Soybean land trade among countries in the year of 2022.

Our data encompassed detailed international trade information for four major food crops - wheat, maize, rice, and soybeans - across 254 countries from 2000 to 2022. Geographical and economic classifications of these countries and regions are provided in SI-Table 1

Table S1. Geographical and economic classifications of these countries and regions.

Country Type	Country
ARG	Argentina
AUS	Australia
BRA	Brazil
CAN	Canada
CHN	China
EGY	Egypt
EU27	Austria
INA	Indonesia
IND	India
IRN	Iran
JAP	Japan
MEX	Mexico
NZL	NewZealand
PAK	Pakistan
RSA	SouthAfrica
RUS	RussianFederation
THA	Thailand
TUK	Türkiye
UKR	Ukraine
USA	UnitedStatesofAmerica

LDC	Afghanistan, Angola, Bangladesh, Benin, Bhutan, BurkinaFaso, Burundi, Cambodia, CentralAfricanRepublic, Chad, Comoros, DemocraticRepublicoftheCongo, Djibouti, Eritrea, Ethiopia, EthiopiaPDR, Gambia, Guinea, Guinea-Bissau, Haiti, Kiribati, LaoPeople'sDemocraticRepublic, Lesotho, Liberia, Madagascar, Malawi, Mali, Mauritania, Mozambique, Myanmar, Nepal, Niger, Rwanda, SaoTomeandPrincipe, Senegal, SierraLeone, SolomonIslands, Somalia, SouthSudan, Sudan, Sudan(former), Timor-Leste, Togo, Tuvalu, Uganda, UnitedRepublicofTanzania, Yemen, Zambia
ROW	Albania, Algeria, Antigua and Barbuda, Armenia, Azerbaijan, Bahamas, Bahrain, Barbados, Belarus, Belize, Bolivia (Plurinational State of), Bosnia and Herzegovina, Botswana, Bouvet Island, Brunei Darussalam, Cabo Verde, Cameroon, Chile, Colombia, Congo, Cook Islands, Costa Rica, Côte d'Ivoire, Cuba, Cyprus, Czechoslovakia, Democratic People's Republic of Korea, Dominica, Dominican Republic, Ecuador, El Salvador, Equatorial Guinea, Eswatini, Faroe Islands, Fiji, French Guiana, French Polynesia, Gabon, Georgia, Ghana, Grenada, Guadeloupe, Guatemala, Guyana, Heard and McDonald Islands, Honduras, Iceland, Iraq, Israel, Jamaica, Johnston Island, Jordan, Kazakhstan, Kenya, Kuwait, Kyrgyzstan, Lebanon, Libya, Malaysia, Maldives, Marshall Islands, Martinique, Mauritius, Micronesia (Federated States of), Midway Island, Monaco, Mongolia, Montenegro, Morocco, Namibia, Nauru, New Caledonia, Nicaragua, Nigeria, Niue, North Macedonia, Norway, Oman, Palestine, Panama, Papua New Guinea, Paraguay, Peru, Philippines, Puerto Rico, Qatar, Republic of Korea, Republic of Moldova, Réunion, Saint Kitts and Nevis, Saint Lucia, Saint Vincent and the Grenadines, Samoa, Saudi Arabia, Serbia, Serbia and Montenegro, Seychelles, Singapore, South Georgia and the South Sandwich Islands, Sri Lanka, Suriname, Svalbard and Jan Mayen Islands, Switzerland, Syrian Arab Republic, Tajikistan, Tokelau, Tonga, Trinidad and Tobago, Tunisia, Turkmenistan, United Arab Emirates, United Kingdom of Great Britain and Northern Ireland, United States Minor Outlying Islands, Uruguay, USSR, Uzbekistan, Vanuatu, Venezuela (Bolivarian Republic of), Viet Nam, Wake Island, Yugoslav SFR, Zimbabwe
